# A Potential Route of Capsaicin to Its Binding Site in the TRPV1 Ion Channel

**DOI:** 10.1021/acs.jcim.1c01441

**Published:** 2022-05-03

**Authors:** Carmen Domene, Leonardo Darré, Victoria Oakes, Saul Gonzalez-Resines

**Affiliations:** † Department of Chemistry, King’s College London, Britannia House, 7 Trinity Street, London SE1 1DB, UK; ‡ Department of Chemistry, University of Bath, 1 South Building, Claverton Down, Bath BA2 7AY, UK; § Chemistry Research Laboratory, University of Oxford, Mansfield Road, Oxford OX1 3TA, UK

## Abstract

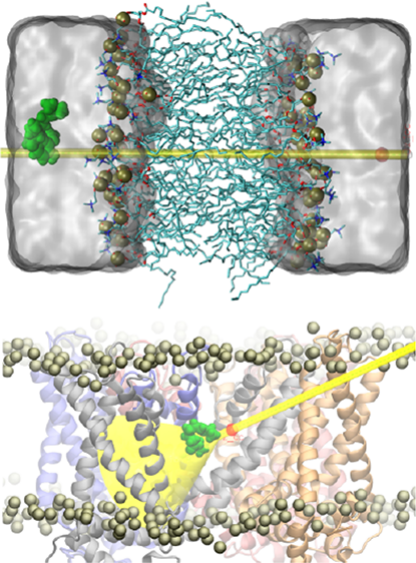

Transient receptor potential (TRP) ion channels are important pharmacological targets because of their role in the perception of pain, and so, understanding their chemical regulation is essential for the development of analgesic drugs. Among the currently known TRP channel chemical agonists, capsaicin, the active compound of chili pepper, is probably the most exhaustively studied. The availability of the three-dimensional structure of the vanilloid receptor 1 (TRPV1) has fueled computational studies revealing the molecular details of capsaicin binding modes. Although this is a significant step, a comprehensible binding mechanism or pathway is invaluable for targeting TRP channels in modern pharmacology. In the present work, free-energy and enhanced sampling techniques have been used to explore a possible membrane-mediated pathway for capsaicin to enter the TRPV1 binding pocket where capsaicin accesses the protein starting at the extracellular milieu through the outer leaflet and into its binding site in the protein. The main states visited along this route have been characterized and include (i) a bound state in agreement with the binding mode “head-down, tail-up” and (ii) an alternative state corresponding to a “head-up, tail-down” binding mode. In agreement with previous reports, binding is mediated by both hydrogen bonds and van der Waals interactions, and residue Y511 is crucial for stabilizing the bound state and during the binding process. Together, these results provide a foundation to further understand TRPV channels, and they could be used to guide therapeutic design of selective inhibitors potentially leading to novel avenues for pharmacological applications targeting the TRPV1 channel.

## Introduction

Transient receptor potential (TRP) ion channels constitute a superfamily of cation channels found at the core of sensory physiology in the animal kingdom. They have a remarkable ability to respond to a wide range of chemical and physical stimuli in excitable and non-excitable cells.^1^ As a consequence of such a broad physiological activity, alterations in their normal activity are associated with several human disorders.^2^ Thus, understanding the molecular details of TRP modulation is fundamental for the development of highly effective therapeutic approaches. In particular, TRPV1 belongs to a class of TRP channels able to respond to environmental temperature changes, thus enabling somatosensory cells to detect noxious temperatures. In addition, the TRPV1 thermal activation threshold (∼43 °C) can be modulated (lowered) by products resulting from tissue damage and inflammation. This makes the TRPV1 channel an essential player in injury-related hyperalgesia and pain hypersensitivity (see Julius^3^ and Carnevale and Rohacs^4^ and references therein). A breakthrough in this area was the resolution of the structure of the vanilloid receptor 1 (TRPV1) under three conditions: (i) without any agonist,^5^ (ii) in the presence of the agonist capsaicin, and (iii) in the presence of the agonists resiniferatoxin (RTX, a vanilloid from *Euphorbia resinifera*) and the spider double-knot toxin (DkTx).^6^ Density in the vanilloid pocket in the capsaicin-bound TRPV1 structure defined at atomic detail an important allosteric regulatory site for inflammatory agents or inverse agonists.^6^ In the closed conformation of TRPV1, the apo state, some electron density was also observed and attributed to a lipid molecule.^5^ The hypothesis proposed was that, in the absence of capsaicin, the binding pocket may be occupied by such a lipid molecule that would compete with capsaicin for the site during activation.

Following the release of these TRPV1 channel structures, cryo-EM structures of TRPV1 in nanodiscs were solved in the apo form and in complex with RTX or capsazepine, with remarkably well-defined electron density.^7^ These structures facilitated identification of the orientation of the ligand and the main contacts with the channel (S512, R557, L515, V518, M547, L669, T550, and I573), which had been hampered by the lower resolution in the original TRPV1 cryo-EM experiments. All these structures lacked the N- and C-termini and the extracellular S5-P-loop. Subsequently, the full-length TRPV1 from squirrels was resolved by cryo-EM providing details about the extracellular cap domain formed by the S5-P-loops and the C-terminus that wraps around the three-stranded β-sheet connecting elements of the TRPV1 intracellular domain.^8^

TRP channels belong to the tetrameric 6-TM superfamily of ion channels. In particular, the TRPV1 structure displays a transmembrane topology like that of voltage-gated ion channels with four subunits arranged around a central permeation pore, and with both, the N- and C- termini of each subunit located intracellularly. Each subunit consists of six transmembrane helices (S1–S6) and a loop–helix domain located between helices S5 and S6. Helices S5 and S6 and the selectivity filter constitute the central pore of the channel, which is flanked by a voltage sensor-like (VS-like) domain formed by the S1–S4 helices. The VS-like domain lacks the positively charged amino acids characteristic of the S4 helix in voltage-sensitive channels.^3^ The structure also contains three additional TRP-specific domains including four ankyrin repeats at the N-terminus, involved in multiple ligand-mediated sensitivity modulation, the P360-V415 linker, conserved among TRPV subtypes, and an extended and kinked interfacial helix following S6 known as the signature “TRP domain” found in many TRP channels.^1^

Although several extracts from plants, *e.g.*, piperine, zingerone, gingerol, or shogaol, and essential oil components, such as rose, thyme, and palmarose, act as TRPV1 agonists,^9^ research into TRPV1 chemical modulation has focused on one particular plant extract, commonly known as “capsaicin”. Capsaicin or (*E*)-*N*-[(4-hydroxy-3-methoxyphenyl)methyl]-8-methylnon-6-en-amide is found in plants of the genus *Capsicum* and is the active component of chili peppers, responsible for the burning sensation experienced upon consumption. Capsaicin was found to interact with a specific and selective receptor in nociceptive neurons,^10^ which was later identified as the TRPV1 ion channel.^11^ Y511 in the S3 helix or T550 in the S4 helix was identified as a key residue in capsaicin binding by exploiting the organism-specific sensitivity to capsaicin by means of chimeric rat (sensitive)/chicken (insensitive) or rat/rabbit (insensitive) TRPV1 constructs.^12,13^ Mutagenesis studies^13^ showed that the binding affinity of capsaicin was substantially reduced in a T550I human mutant and rat TRPV1 channels, confirming the role of T550. Initial structural studies failed to provide sufficient detail to reveal precisely how vanilloids bind to TRPV1. In another study,^12^ capsaicin sensitivity was eliminated using S512Y and Y511A mutations, which are located at the intracellular end of S3 in rat TRPV1, suggesting that capsaicin may bind in the vicinity of these residues. Chu *et al.*(14) also showed that chimeras containing rat E570-V686 swapped in chicken receptors displayed capsaicin sensitivity. In particular, the A578E mutation in the S4–S5 helix of the chicken receptor enhanced capsaicin sensitivity in the micromolar range. Substitution of 578E by lysine, glutamine, or proline equally elicited capsaicin sensitivity in chicken TRPV1. Replacing the corresponding rat TRPV1 residue E570 with lysine or glutamine retained capsaicin sensitivity. A hydrophilic capsaicin analogue was found to activate a chicken TRPV1-A578E mutant.^14^ However, zingerone, a hydrophilic vanilloid agonist, did not activate any A578 mutants. These observations suggest that (i) A578 may participate in vanilloid binding, but the vanilloid group alone is not sufficient to trigger activation and (ii) that minimal analogous changes of TRPV1 in different species alter capsaicin responses. Based on mutagenesis experiments, two alternative capsaicin binding-modes were proposed. In the suggested “head-down, tail-up” orientation, the vanillyl ring points downward and interacts with the aromatic ring of Y511.^7^ This mode was validated by several computational approaches.^15−17^ In ref (15), capsaicin and a library of capsaicin analogs were docked to TRPV1 structures using the electron density observed in the cryo-structures as a guide for the computational work, and mutagenesis studies supported the computational predictions. By means of umbrella sampling calculations, Hanson *et al.*(18) showed that capsaicin prefers to be localized at an interfacial region of the lipid membrane and that it is likely to flip from the extracellular to intracellular leaflet of the membrane to access the binding pocket. By a combination of experimental and computational work, Yang *et al.*(17) suggested that, inside the binding pocket, capsaicin adopts a head-down, tail-up configuration and its aliphatic tail interacts with the channel through nonspecific interactions.

In the alternative “head-up, tail-down” mode, capsaicin adopts the inverted orientation where the aliphatic tail points toward Y511 and the vanillyl ring points upward and interacts with T550 and W549.^9^ In agreement with this, the structural studies responsible for the first cryo-EM TRPV1 structure^5,6^ assigned electron density to capsaicin in a pocket involving residues from the S3 (Y511) and S4 (M547 and T550) helices.^5,6^ Further contacts were also identified including E570 in the S4S5-linker and L669 in the S6 helix of an adjacent subunit. However, the resolution of the structure (4.2 Å) was insufficient to resolve the orientation of capsaicin in the binding site, precluding a full characterization of its binding mode. Previously, a combination of docking calculations and functional experiments endorsed a head-down, tail-up conformation.^13^ Such a binding pose would require non-specific hydrophobic contacts of the aliphatic tail with the protein (*e.g.*, F543) and hydrogen-bond interactions between the amide moiety and T550. Additionally, a mechanism for ligand-induced activation has been proposed involving a hydrogen bond between the vanillyl ring and residue E570.^7^ From an entirely computational study using molecular docking and unbiased and biased molecular dynamics (MD) simulations, including free energy perturbation and metadynamics, a structural model of the capsaicin–channel complex was proposed.^16^ In addition, the standard free energy of binding was estimated using alchemical transformations coupled with conformational, translational, and orientational restraints on the ligand. Key binding modes consistent with previous experimental data were identified, and subtle, but essential dynamical features of the binding site were characterized.^16^ Finally, the information contained in the experimental electron density maps was used to accurately determine the binding mode of capsaicin and resiniferatoxin, a phorbol ester isolated from the irritant lattices of the Moroccan cactus. The computational results were validated by mutagenesis.^15^

Pharmaceutical companies have shown an enormous interest in TRP channel-modulator drug discovery programs because TPR channels detect noxious stimuli, and in this sense, targeting the initial stages of the pain pathway should be a relevant strategy for developing novel analgesics, especially when studies had shown that capsaicin desensitization inhibited pain dramatically in animal models. However, such animal models were found to be poor predictors of clinical efficacy, and in light of this, pharmacotherapy has turned out to be guided by human genetics.^19^ Likewise, drug discovery programs have not succeeded in finding new therapies, and clinical trials have consistently failed as a result of the severe side effects the selected compounds inflict.^4^ Although substantial work has been invested into exploiting capsaicin and its analogs for pharmaceutical purposes, and recent advances in cryo-EM have resulted in unprecedented and instrumental structural information at atomic resolution, structural studies alone do not provide the full picture of the dynamical processes under consideration and, unfortunately, do not reveal details of the evolution of the interactions between the protein and its agonists during activation. In this respect, the work described in this manuscript contributes to our knowledge about the route capsaicin takes to bind to its target and serves as a model to understand many other naturally occurring capsaicinoids with similar chemical features. Currently, limited information is available regarding how capsaicin accesses the known TRPV1 binding pocket, in particular, if capsaicin directly enters the binding site through the protein or if it reaches the binding site via the membrane. A membrane-mediated pathway is supported by comparison of MD simulations of the voltage sensor-like domain in a lipid bilayer and the translocation potential of mean force of capsaicin across the membrane.^18^ However, full characterization of the pathway by which capsaicin approaches its binding site has not yet been described. In the present study, funnel metadynamics simulations extending over 6 μs were used to evaluate the prospect of a membrane-mediated pathway providing in turn the structural details for such a scenario. Together, these results provide a foundation to further understand activation and modulation of TRPV1 and could be used to guide therapeutic design of selective inhibitors potentially leading to novel avenues for pharmacological applications targeting this channel family.

## Computational Methods

### Membrane Permeation

The starting system was a pre-equilibrated POPC (1-palmitoyl-2-oleoyl-*sn*-glycero-3-phosphocholine) bilayer that was solvated using the Solvate plug-in of VMD.^20^ The CHARMM36 force field^21^ was used to describe the lipids, and the TIP3P model^22^ was used to describe water. Initial steric clashes were removed by 5000 steps of minimization and 0.5 ns of equilibration with restraints on the phosphate groups to maintain the thickness of the bilayer during the relaxation/thermalization of the system followed. A capsaicin molecule with parameters specified in ref (16) was then added in the water phase, and the system was further equilibrated for another 0.5 ns. Both the equilibration and the biased MD production were performed in the NpT ensemble, with a semi-isotropic pressure coupling at 1 atm using the Nose–Hoover Langevin piston.^23^ The temperature was controlled at 310 K by means of the Langevin thermostat. Long-range electrostatic interactions were treated using the particle mesh Ewald algorithm,^24^ and van der Waals forces were smoothly switched off, using the vdW force switching option, between 10 and 12 Å. The RATTLE algorithm^25^ was used to constrain all bonds involving hydrogen atoms to use a time step of 2 fs. The multi-time step algorithm r-RESPA^26^ was used to integrate the equations of motion. Non-bonded short- and long-range forces were computed at every time step.

**Figure 1:**
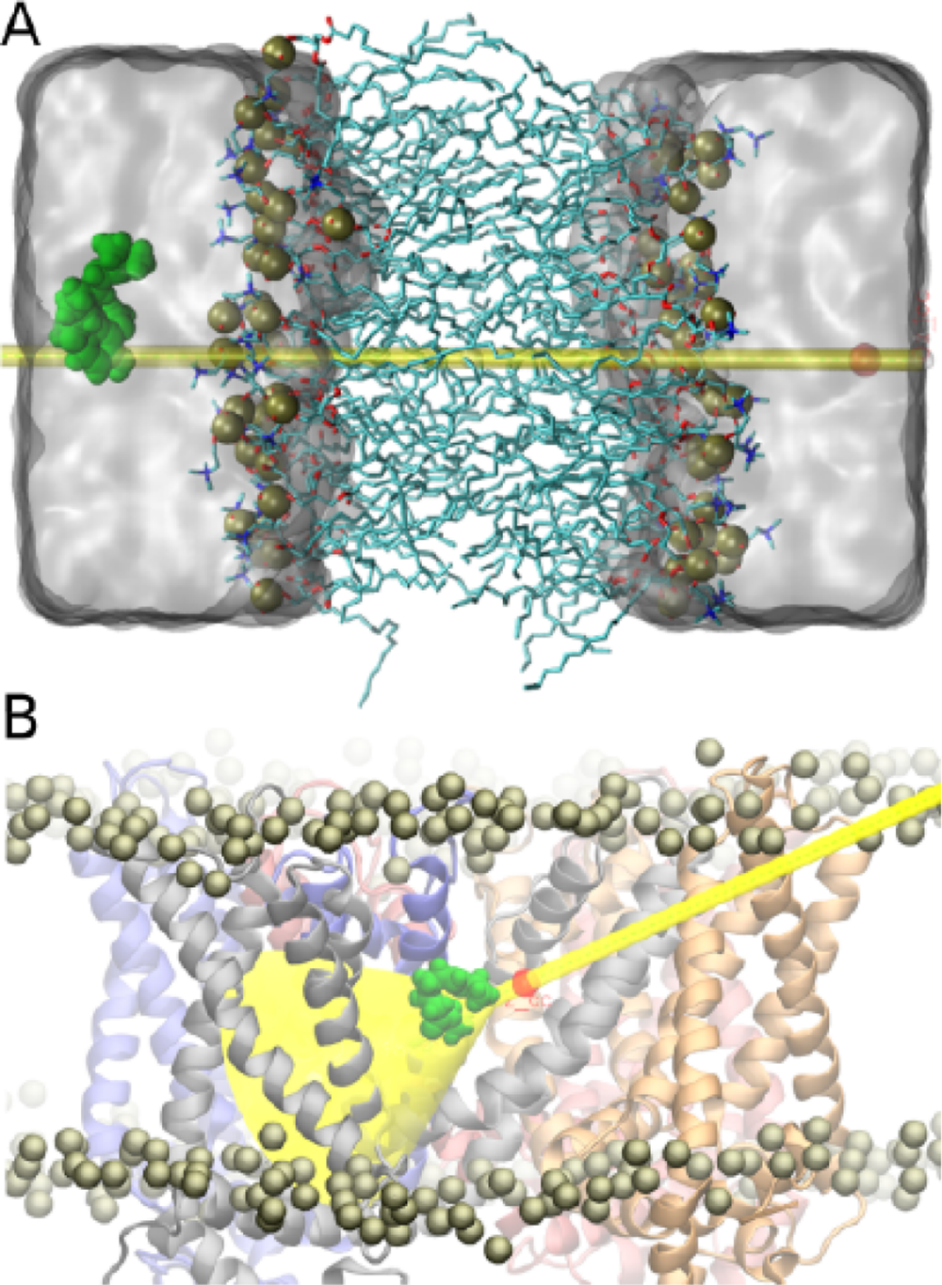
Systems. (A) Initial configuration of the membrane permeation setup showing the POPC lipid bilayer (licorice representation; phosphate atoms are indicated with brown vdW spheres), the aqueous environment (gray surface representation), and capsaicin molecule (green vdW sphere representation). The space restraint applied to the center of mass of the vanillyl ring is indicated by the transparent cylinder. The axis of the latter was used to define the progression collective variable biased during the metadynamics simulation. (B) Representative configuration of the funnel metadynamics simulation setup. The trans-membrane domain of TRPV1 is shown colored by the chain identifier (chains A: blue, B: red, C: gray, and D: orange). POPC phosphate atoms and the capsaicin molecule subject to the metadynamics biasing potential are indicated with brown and green vdW spheres, respectively. The space restraint applied to the center of mass of the vanillyl ring is indicated by the transparent funnel cylinder. The axis of the latter was used to define the progression and radial distance collective variables biased during the metadynamics simulation.

Permeation of capsaicin across a POPC lipid bilayer was studied by means of well-tempered metadynamics simulations. In this method, a history-dependent biasing potential acting on a set of collective variables, which are functions of the particle positions that are used to describe the progress along a reaction pathway, is constructed from the deposition of Gaussians with decreasing height as the simulation proceeds. Application of such bias favors the sampling of regions of the collective variable space difficult to access in normal MD simulations. To study the permeation process, the distance between a reference point in the water phase and the center of mass of the capsaicin vanillyl ring was used as the collective variable. To focus the sampling on the direction orthogonal to the membrane (*z* axis), a 2 Å diameter cylinder-shaped restraint oriented along the *z* axis was applied on the capsaicin vanillyl ring center of mass (Figure 1A). Gaussians with an initial height of 0.1 kcal·mol^–1^ and width of 0.25 Å were deposited every 2 ps, with a bias factor of 10. The total simulation time employed to achieve convergence was over 2.5 μs.

### Transport of Capsaicin from the Membrane to the TRPV1 Binding Pocket

Experimentally, it has been shown that capsaicin can activate TRPV1 when applied from either side of the membrane, although there are other reports at odds with this. To explore the conformational landscape underlying the access of capsaicin from the membrane to the protein binding pocket, funnel metadynamics simulations were employed. This variant of the metadynamics technique uses a funnel-like spatial restraint to favor the sampling of the binding pocket and its surroundings as well as the entry pathway reducing the computational costs associated with sampling irrelevant areas of the system. To ensure full sampling of the desired space, two collective variables are used: (i) the progression along the funnel axis, defined as the position of the center of mass of the vanillyl ring projected on the funnel axis, and (ii) the radial distance of the center of mass of the vanillyl ring to the funnel axis. The initial structure used for the funnel metadynamics simulation was taken from a representative snapshot of an unbiased MD simulation of the system reported elsewhere.^16^ This system consisted of the transmembrane (TM) domain (residues: 394–719) of the cryo-EM structure of the TRPV1 solved in the presence of capsaicin (PDB ID: 3J5R). The protein was embedded in a lipid bilayer (490 POPC molecules) aligned to the *x*-*y* plane of a rectangular box filled with water and ions (NaCl 150 mM). The system contained four capsaicin molecules inserted in their binding pockets using docking calculations.^16^ In the present work, only one of the four capsaicin molecules was subject to the biasing potential of the funnel metadynamics. Figure 1B depicts the specific setup for the TRPV1 capsaicin system. The CHARMM22 force field with the CMAP^27^ correction was used for the protein, CHARMM36^21^ was used for the lipids, the TIP3P model^22^ was used for water, and the standard CHARMM and NBFIX parameters^28^ were used for ions. A total simulation time of ∼2.7 μs was generated. Gaussians with an initial height of 0.1 kcal·mol^–1^ and sigma values of 0.25 and 0.15 Å for the progression and radial distance collective variables, respectively, were deposited every 2 ps, with a bias factor of 10. Despite the length of the run, convergence of this simulation was not achieved due to the choice of parameters. An additional simulation, using the same funnel bias, Gaussians with an initial height of 0.1 kcal·mol^–1^ and sigma values of 0.25 and 0.15 Å for the progression and radial distance collective variables, respectively, deposited every 2 ps, with a bias factor of 15, was also performed, and convergence was achieved with a total simulation time of ∼1.1 μs. Following the protocol described in ref (29), a block averaging algorithm was employed to compute the statistical error of the free energy from the biased simulation related to the simulation length. The values obtained are 2.51, 2.38, and 2.75 kcal/mol filtering with 3, 4, or 5 kcal/mol. All trajectories reported here were generated using NAMD2.8^30^ patched with PLUMED 1.3.^31^ The free energy as a function of the collective variables biased was constructed using the sum_hills utility of PLUMED 1.3. Snapshots corresponding to each free energy basin were extracted using the driver utility of PLUMED 1.3, which were further clustered using the GROMACS^32^ g_cluster tool with a linkage algorithm and an RMSD cutoff of 1 Å.

### Calculation of Standard Free Energy of Binding of Capsaicin in the Head-Up, Tail-Down Pose

The initial starting structure of capsaicin in the “up” conformation in the binding site was obtained from molecular docking using AutoDock4.0^33^ following a protocol identical to that described in ref (16). The docking calculations were performed in one out of the four possible binding sites, that is, one per interface between adjacent monomers. Subsequently, the best docking pose was replicated in all four binding sites, and the resulting system was refined by MD simulations. The channel that was inserted in a pre-equilibrated lipid bilayer of 1-palmitoyl-2-oleoyl-*sn*-glycero-3-phosphocholine (POPC) molecules was used and then solvated using the Solvate plug-in.^20^ Identical force field parameters to those described in the previous section were employed. A 5000-step minimization was followed by a sequence of three 0.5 ns equilibration runs with constraints applied initially on the lipid head groups, capsaicin molecules, and all protein atoms, subsequently only on all protein atoms and, finally, only the protein backbone. A Langevin thermostat was employed to maintain the temperature at 310 K, and the Nose–Hoover Langevin piston^23^ maintained a semi-isotropic pressure at 1 atm. The particle mesh Ewald algorithm^24^ was used compute the long-range electrostatic interactions, and van der Waals interactions were smoothly switched off using a switching function between 10 and 12 Å. All bonds involving hydrogen atoms were constraint by means of the RATTLE algorithm^25^ to use a time step of 2 fs. An initial trajectory of 100 ns was evolved using NAMD2.9. Subsequently, the standard free energy of binding of capsaicin to TRPV1 in the head-up, tail-down pose was predicted using a protocol described by Gumbart *et al.*(34) and already employed by us to calculate the standard free energy of binding of capsaicin in the head-down, tail-up pose.^16^ Convergence of the alchemical steps was evaluated by estimating the overlap of the difference in free energy distributions for each window in the forward and backward directions. The Bennett acceptance ratio (BAR)^35^ was used to estimate the difference in free energy as implemented in the ParseFEP tool^36^ in VMD.^20^

## Results

### Permeation of Capsaicin across the Membrane

**Figure 2:**
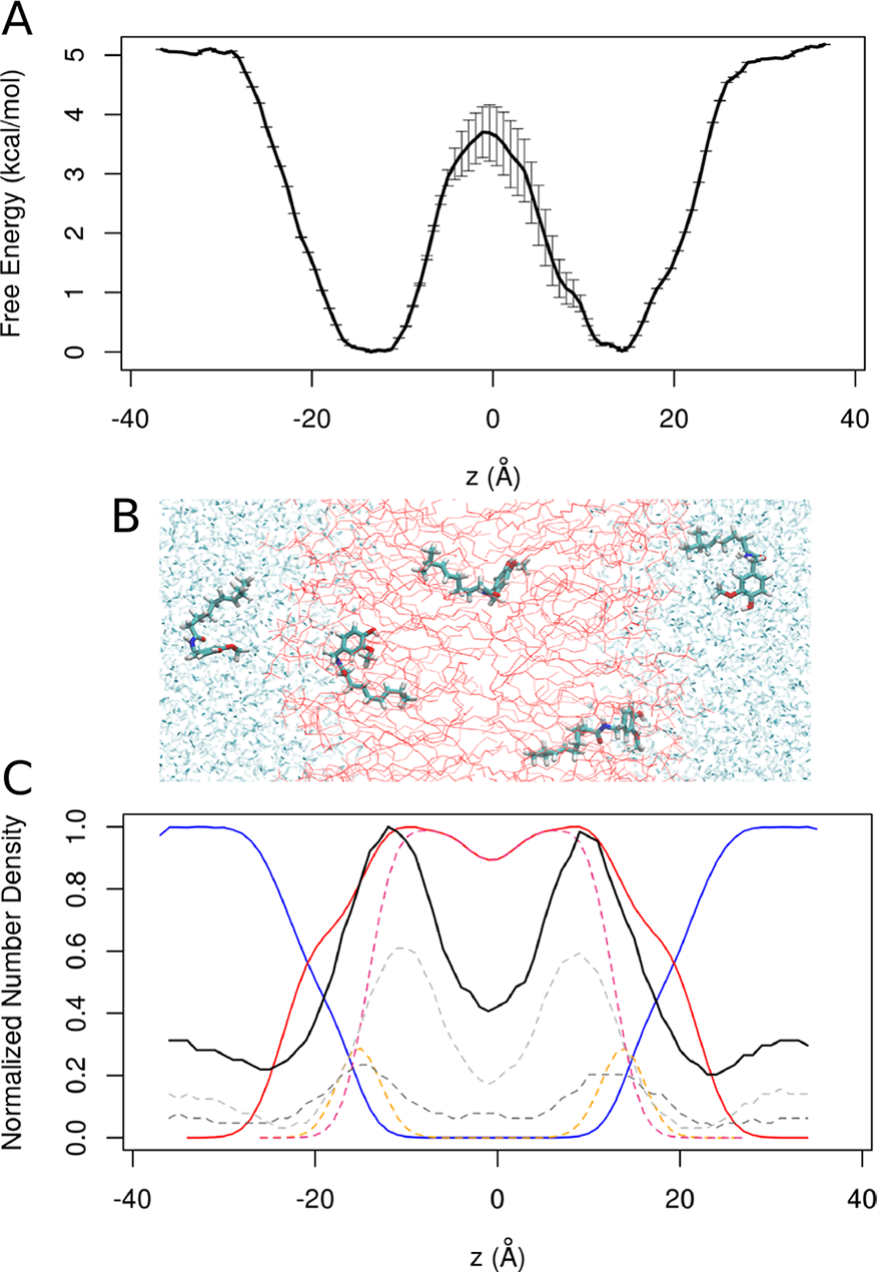
Free energy and structure of capsaicin during membrane permeation. (A) Average and standard deviation of the potential of mean force (PMF) associated with the translocation of capsaicin across a POPC lipid bilayer calculated from the PMF profiles obtained from the sum of Gaussian kernels deposited after 2.42, 2.44, 2.46, 2.48, and 2.5 μs of well-tempered metadynamics simulation. (B) Representative structures of capsaicin during the permeation process taken from simulation frames corresponding to *z*-axis windows of 3 Å centered at −32, −14, 0, 14, and 32 Å (from left to right). (C) Normalized number density profiles of water, POPC lipids, and capsaicin (blue, red, and black lines, respectively) along the *z* axis. Also shown are the contribution of the carbonyl groups and lipid tails to the POPC density (orange and violet dotted lines, respectively) and the decomposition of capsaicin density in two components, the vanillyl ring and the peptidic bond, and the hydrophobic tail (dark and light gray dotted line, respectively).

Two pathways have been proposed in the literature describing how capsaicin reaches its binding site in TRPV1. The first one involves direct translocation to the binding site from the aqueous phase, while the second involves accessing the site from the lipid phase after translocation through the membrane starting at the aqueous solution. The former could be supported by mutagenesis experiments, while the latter has received support from umbrella sampling calculations^18^ and is in line with the capsaicin octanol/water partition coefficient^37,38^ (log *P*^o/w^ ∼3.8, *i.e.*, Δ*G*_O-W_ ∼ +5 kcal·mol^–1^). In this study, to begin with, the permeation process of capsaicin across a POPC bilayer was investigated and compared to previous reports using the modeling approach described earlier. The calculated potential of mean force (PMF) for capsaicin translocation through the lipid bilayer, along with representative structures of capsaicin in the aqueous and membrane environments, is shown in Figure 2.

Two iso-energetic minima inside the membrane are observed for capsaicin that correspond to the position of POPC carbonyl groups from each leaflet, separated by a maximum at the position of the lipid tails (Figure 2A,C). This is consistent with the amphipathic character of capsaicin; capsaicin is stabilized within the membrane by interactions of its polar amide bond and methoxy and hydroxyl groups with the POPC carbonyl groups, while its hydrophobic tail points toward the POPC lipid tails (Figure 2C). One of the aims of the analysis was to obtain the average orientation of capsaicin from its head and tail number density profiles relative to the membrane component profile; the head group of capsaicin (dark gray dashed line) overlaps with the POPC carbonyl density (orange dashed line), and capsaicin tail density (light gray dashed line) appears closer to the membrane core and overlaps with the POPC tail density (purple dashed line). Qualitatively, the orientation of capsaicin is shown in Figure 2B. Regarding the error bars of the free energy profile, it appears that, at the core of the bilayer, the error is higher than the rest of the free energy profile values albeit rather small (∼0.5 kcal·mol^–1^). In agreement with the octanol/water partition coefficient of capsaicin, the free energy difference between capsaicin in the aqueous solution and within the membrane accounts for ∼5 kcal·mol^–1^, confirming that the residence in the membrane is thermodynamically favored relative to the solvent. Migration from one leaflet of the membrane to the other requires overcoming a free energy barrier of ∼3.5 kcal·mol^–1^, in good agreement with a previous report.^18^ However, it is not clear whether capsaicin will penetrate the cell membrane and then access the protein binding site or if, alternatively, it will penetrate the cell membrane, reach the cytosol, and, subsequently, interact with the cytosolic side of the protein to access the binding site. A third scenario could be envisaged, where capsaicin directly penetrates the protein until it reaches its binding site.

### Capsaicin Pathway from the Membrane to the TRPV1 Pocket

**Figure 3:**
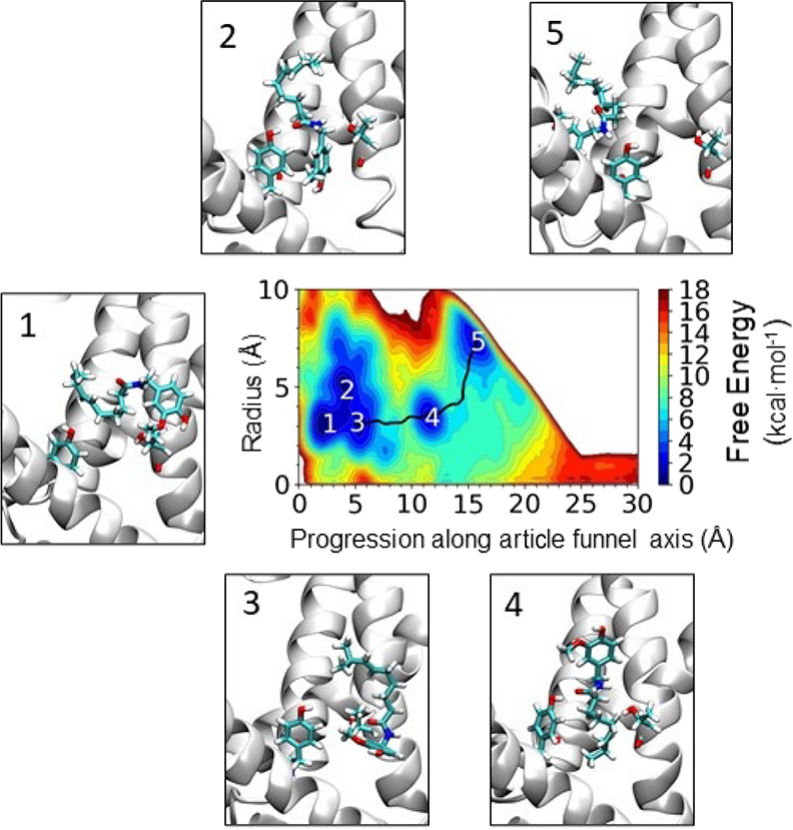
Conformational landscape of capsaicin along the pathway from the membrane to the TRPV1 binding site. Probability density distribution obtained from the funnel shape-restrained metadynamics simulation, indicating the regions of highest probability to find capsaicin (regions 1–5). The black line corresponds to the minimum energy path (MEP) connecting the various minima present in the potential of mean force and calculated using the string method described in ref (39). The biased potential of metadynamics was used to force capsaicin to move both parallel and orthogonal to the funnel axis while restraining its position within the funnel space. Representative structures of capsaicin from high probability regions in the framework of the binding pocket are shown obtained using the same orientation of the protein in each snapshot. Only one chain of the protein is shown in cartoon representation in white and selected residues in licorice representation (Y511 and T550). Capsaicin is shown in licorice representation.

To study the potential pathways that capsaicin might take to reach its binding site in the TRPV1 channel from the lipid membrane, well-tempered metadynamics simulations with an applied funnel-shaped restraint were performed. The biased potential of metadynamics was used to force capsaicin to move both parallel and orthogonal to the funnel axis (“progression” and “radius” axis in Figure 3) while restraining its position within the funnel space. Consequently, if the position of the funnel is chosen wisely, then the progression of capsaicin from the membrane to the binding pocket and back again can be explored exhaustively. Although free energy estimations are possible using this technique, in the first attempt, after 2.7 μs of simulation, deposition of Gaussians of a height of ∼0.08 kcal·mol^–1^ (80% of maximum height) was still observed, indicating that convergence was not achieved and precluding quantitative free energy estimates. In this case, the hill height chosen was too small, and it was unlikely that it would ever get near 0%. Nevertheless, identification of the most probable regions within the funnel allowed characterization of a binding route featuring a bound state in agreement with previous reports,^12^ an intermediate state suggested by mutagenesis experiments^13^ and docking calculations,^16^ and two additional states that correspond to the initial encounter of capsaicin with the protein from within the membrane. The PMF of capsaicin in the two-dimensional space defined by the directions parallel (“progression”) and orthogonal (“radius”) to the funnel axis is shown in Figure 3. Free energy minima are observed at progression values of 2, 4, 5, 12, and 17 Å (Figure 3). Representative structures of capsaicin in the binding pocket are shown for the converged metadynamics run (and in Figure S1 for the non-converged metadynamics run).

Initially, capsaicin at the membrane core (Figure 3 region 5) is attracted toward the entry of the binding pocket, and subsequently, it reaches an intermediate position (region 4) characterized by a head-up, tail-down orientation, resembling the alternative binding conformation previously suggested by mutagenesis studies^12,17^ and docking calculations.^16,17^ The last state in the pathway (regions 2 and 3) corresponds to a head-down, tail-up orientation, consistent with the currently accepted bound mode of capsaicin to the TRPV1 channel^13^ before it returns to the membrane core. The free energy barrier required to arrive at the intermediate state from the region located at the entry of the binding pocket in the intermediate state (from minimum 5 to minimum 4) was found to be 5.8 kcal·mol^–1^ and from the intermediate state to the bound state was 6.9 kcal·mol^–1^ (minimum 4 to minimum 3).

In a previous study, we presented extensive work on the thermodynamics governing the binding of capsaicin to TRPV1 by means of molecular docking and unbiased and biased MD simulations, including free energy perturbation and metadynamics. In good agreement with experimental reports, capsaicin was found to bind in a head-down, tail-up conformation where the vanillyl ring points toward the S4S5-linker and its lipid tail points upward in the direction of the S4 helix. Four minima corresponding to direct or water-mediated interactions between the amide moiety of capsaicin and residues Y511/T550 of the protein differ by energy differences comparable to a single hydrogen bond. In addition, the standard free energy of binding from the bulk solution to one of these basins was reported to be −10.6 ± 1.7 kcal·mol^–1^. In the present study, if we were to describe the energetics of the transport via the full path from the aqueous solution to the binding site via the cell membrane, we have already recorded the value of the free energy difference between capsaicin in the aqueous solution and within the membrane that accounts for ∼5 kcal·mol^–1^ and migration from one leaflet of the membrane to the other requires overcoming a free energy barrier of ∼3.5 kcal·mol^–1^. In addition, once close to the binding site and still embedded in the membrane, the free energy required to arrive at the intermediate state is 5.8 kcal·mol^–1^ (corresponding to the “up” conformation) and from the intermediate state to the bound state is 6.9 kcal·mol^–1^ (corresponding to the “down” conformation). To further interpret the data from the various metadynamics runs undertaken now, we extended our previous study and calculated, using the same protocol, the standard free energy of binding of capsaicin to TRPV1 in the head-down, tail-up pose, which was found to be −12.4 ± 1.4 kcal·mol^–1^. In qualitative terms, these values are consistent with a value of −10.6 ± 1.7 kcal·mol^–1^ reported in our previous study for the free energy required to take capsaicin from the aqueous solution to the TRPV1 pocket in the pose corresponding to head-up, tail-down conformation and with the ones reported from the funnel metadynamics: Entry of the binding pocket in the head-down, tail-up conformation is 5.8 kcal·mol^–1^ and from the head-down, tail-up to the head-up, tail-down conformation accounts for 6.9 kcal·mol^–1^. Unfortunately, comparison to experimental data is not currently possible as the dissociation constant for capsaicin is not experimentally available but the decomposition of the pathway in energetic steps provides insightful information.

Principal component (PC) analysis of the position of capsaicin in the structurally aligned binding site followed by hierarchical clustering in the PC space^40^ in each high probability region was used to portrait representative configurations of capsaicin. It was found that the degree of structural heterogeneity within each cluster depends on the region considered; in both funnel metadynamics runs, the same conformations were sampled. In the converged metadynamics run, the region located at the entry of the binding pocket shows three principal clusters with populations of (A:60%, B:23%, C:17%) of the total snapshots (region 5 in Figure 3). The intermediate state in region 4 presents three main clusters with populations of (A:67%, B:20%, C:13%) of the total snapshots from region 4. Two states of comparable energy characterize the bound state in regions 2 and 3 with principal clusters of populations (A:73%, B:15%, C:12%) and (A:11%, B:57%, C:32%) of the total snapshots for each region respectively. Finally, region 1 is characterized by clusters composed of populations (A:75%, B:13%, C:12%).

Table 1 shows information about interactions of capsaicin and the protein, and the orientation of capsaicin with respect to the side chains of Y511 and T550 residues is illustrated in Figure 3 for the converged metadynamics run (and in Figure S1 for the not converged run) for each of the most populated clusters in all the high density regions. Clusters are denoted using the number of the region it is associated to and the cluster population, *i.e.*, a cluster with 75% population in region 1 is referred as 1_75%_. The main difference between the clusters observed in region 1 is the orientation and contacts of the aliphatic tail, *e.g.*, in cluster 1_75%_, the tail is oriented toward the S4S5-linker, and in cluster 1_13%_, the tail is folded against the vanillyl ring. This suggests a binding mechanism in which hydrophobic residues in helices S3 and S4 anchor capsaicin vanillyl and amide groups, respectively, allowing the tail to flip between the outside and inside of the binding pocket. Once the tail is inside the pocket (cluster 1_13%_), further rearrangements lead to the intermediate state. Like in region 1, in region 5, two protein anchoring points fix the position of the vanillyl and amide groups, allowing the aliphatic tail to explore the binding site region until it adopts a position like that of cluster 4. However, in this case, the vanillyl ring interacts with the S4S5-linker (*i.e.*, I573 and L575) and Y511 forms hydrogen bonds not only with the amide group but also with the methoxy moiety. This reflects an alternative binding route based on the same principle described for region 1, *i.e.*, anchoring of the capsaicin vanillyl and amide groups, while the aliphatic tail searches the binding site.

**Table 1:** Interactions between Capsaicin and the Protein in the Most Populated Clusters Obtained from a Principal Component Analysis Followed by Hierarchical Clustering in the PC Space^40^^a^

cluster	capsaicin orientation	protein interactions with capsaicin
1_75_		Phe543, Ala546, Met547, Phe587, Phe591, Leu669
2_73_	head-down, tail up	Thr550, Tyr511, Leu515, Met547, Leu569, E570, Ile661, Leu662, Ala665, Leu673
3_57_	head-down, tail up	Trp549, Thr550, Leu553, Ile573, Val583, Phe587, Gly590, Leu662, Leu 669
4_67_	head-up, tail-down	Ser512, Tyr511, Leu515, Ala546, Met547, Thr550, Glu570, Ile661, Leu662, Leu669
5_60_		Tyr511, Leu515, Val518, Met547, Ile573, Leu574, Leu575

a Clusters are denoted using the number of the region and the percentage population of such a cluster.

In summary, these results indicate a binding mechanism in which two anchoring points are available for capsaicin in the protein: a hydrophobic interaction with S3/S4 or the S4S5-linker and a hydrogen bond with residue Y511. Through these interactions, the capsaicin vanillyl and amide moieties are locked, facilitating the aliphatic tail of capsaicin to explore the binding site. The tail can adopt a position like that in the intermediate state. After the aliphatic tail is positioned, further rearrangements would be required to move the vanillyl ring toward S3/S4 helices to allow capsaicin accessing the intermediate state (transitions from 1 and 5 to 4 in Figure 3). The last step in the binding process involves the inversion of the positions of the vanillyl ring and the aliphatic tail leading to the bound state (transition from region 4 to regions 2 and 3). As observed in some of the cryo-EM structures, the vanilloid head group can be involved in the formation of a salt bridge with E570, an interaction described to contribute to make the S1–S4 and the linker domains a single rigid unit.^17^

## Conclusions

Free-energy based methods are efficient tools widely used to compute the mode of action and the free energy profile associated with the binding of small molecules to proteins. They are based on conformational sampling and exploitation of the full flexibility of the target protein and the ligand as well as allowing considering solvent effects explicitly. In a previous publication, we explored the interaction of capsaicin with the TRPV1 channel by means of docking and we confirmed the experimental observations that had predicted two models of binding: (i) one model where the vanillyl ring would point downward, interacting with the aromatic ring of Y511,^12^ and (ii) a second model where the capsaicin molecule would adopt an inverted orientation.^13^ Subsequently, the standard free energy of binding of the most favorable configuration, the head-down, tail-up, was estimated using alchemical transformations coupled with conformational, translational, and orientational restraints on the ligand, and subtle but essential dynamical features of the binding site were characterized using metadynamics. In this article, we have extended our previous calculations, and the standard binding free energy calculation of a head-up, tail-down capsaicin configuration is presented using the same methodology to complement metadynamics investigations to characterize how capsaicin may travel from the solution to the inside of the lipid membrane and reach its binding site at the core of the protein. Here, we assumed that capsaicin would penetrate the cell membrane, and once inside the membrane core, it would reach the binding site already reported by mutagenesis and cryo-EM structures. Using the well-tempered metadynamics method, which can overcome energy barriers and allow the reconstruction of free energy surfaces (FES), we investigated the translocation of capsaicin from the aqueous solution to the core of the lipid membrane, which was found to be 5.0 kcal·mol^–1^. The FES was reconstructed as a function of a collective variable, the position of capsaicin along the axis perpendicular to the bilayer. It was also found that migration from one leaflet of the membrane to the other requires overcoming a free energy barrier of ∼3.5 kcal·mol^–1^. The next step in our process, the penetration of the agonist from the core of the membrane to its binding site, was studied using funnel metadynamics, where a funnel-shaped restraint potential was applied to reveal the ligand binding mode and accurately calculate the absolute ligand–protein binding free energy. Despite an initial failure, careful selection of the different parameters led to a well-converged free-energy surface. Changes observed in this free-energy landscape correspond to local changes in the network of electrostatic, hydrogen-bond, and hydrophobic interactions between capsaicin, key residues in the protein, and water molecules. In this second step, the barrier to the entrance was calculated to be ∼5.8 kcal·mol^–1^, rendering capsaicin in the head-down, tail-up conformation. The last step corresponds to the rearrangement of the ligand in the binding site to further adopt the “up” conformation with an additional cost of 6.9 kcal·mol^–1^. Considering the present results, a potential route for capsaicin to reach the binding site observed in the cryo-EM structures, a process fundamental for rational drug design, has been rationalized in energetics terms.

Understanding the binding modes of known agonists and antagonists to TRPV1 would significantly contribute to the success of TRPV1 modulator drug design programs.^15^ Despite the efficacy of antagonists such as capsazepine, their effectiveness appears to be dependent on the species tested,^41^ thus limiting the extrapolation of data from animal models to humans that would not be not straightforward. Despite these differences and limitations, more information about the way by which capsaicin reaches its binding site aid in the understanding of the mechanisms controlling TRPV1 activity. A potential membrane-mediated pathway for capsaicin to enter its binding site in the TRPV1 channel from the solvent has been studied using multimicrosecond MD simulations, providing an energetically favorable route and information about the accompanying structural details of this process. Initially, the process of translocation through the membrane was explored using enhanced sampling calculations amounting ∼2.5 μs, obtaining thermodynamic information in agreement with the octanol/water partition coefficient, and theoretical reports on the free energy barrier of capsaicin flipping between the leaflets of the cell membrane. Subsequently, translocation toward the binding site was simulated using combined ∼3.8 μs long funnel metadynamics simulations. The results illustrate a binding mechanism that can be decomposed in three steps. In the first one, the head of capsaicin (vanillyl and amide groups) is anchored to the binding site entry region and the aliphatic tail can sample the pocket. In the second step, once the tail has positioned in the binding site, further rearrangements lead to an intermediate state (head-up, tail-down conformation). Finally, inversion of the position of the vanillyl ring and the aliphatic tail leads to a bound state, which resembles the tail-up, head-down binding mode. Validation of this model would be possible by mutating key residues described in this study, which would alter either the kinetics of binding or the binding affinity of capsaicin. It is conceivable to imagine alternative routes for capsaicin to arrive at its binding site to the one presented here; for instance, capsaicin could penetrate the cell membrane and reach the cytosol and, subsequently, interact with the cytosolic side of the protein to access the binding site. Likewise, capsaicin may interact directly with the protein and reach its final binding site. The effects of some of the most common single and double mutations on the activation by the capsaicin free-energy landscape of the TRPV1 channel could be also exploited in the future by using massive molecular dynamics simulations together with enhanced sampling techniques like the ones employed in this study. Although the biological effects of some of these mutations are clear, a mechanistic explanation linking the mutations to changes in the conformational free-energy landscape and activity of the channel is still missing.

The bilayer composition is known to be important in the regulation of TRPV1 with experimental evidence recording the effect of cholesterol^42^ and PIP_2_.^43^ Thus, in this respect, a limitation of this study is the use of a simple single-component model bilayer, which fails to consider the influence of membrane asymmetry, its rich composition, and thus, overall, the complexity of the real cell membrane. Likewise, we have used a truncated model that facilitated extensive sampling, prohibited otherwise if we had employed the full-length protein. However, it is known that residues in the N- and C-termini are required for capsaicin and RTX activation of TRPV1;^8^ therefore, further simulations of capsaicin interactions with the full-length protein will be needed if a complete picture of the mechanism of activation is desirable.

The intracellular domain of TRPV channels is a highly conserved region adjacent to the internal gate and essential for tetramerization and allosteric activation.^44^ Allosteric modulators of TRPV1 activity are a class of non-competitive antagonists, which interfere with the allosteric mechanism that gates the channel, while uncompetitive antagonists act as open channel blockers preferentially binding to over-activated channels with minimal interaction with the physiologically working channels.^45^ The atomic detailed information provided in this study constitutes a fundamental step to understand TRPV1 chemical modulation at least for those compounds similar to capsaicin, which is at the core of TRP channel drug design.

## Supporting Information Available

Conformational landscape of capsaicin along the pathway from the membrane to the TRPV1 binding site for the simulation (PDF)

## Notes

Requirements for existing software apply to this manuscript. Specific open access software from third parties was used: NAMD2.8 (https://www.ks.uiuc.edu/Research/namd/2.13/features.html), PLUMED 1.3 (https://www.plumed.org/), VMD1.9 (http://www.ks.uiuc.edu/Research/vmd/), Gromacs (https://www.gromacs.org/), AutoDock4.0 (https://autodock.scripps.edu/), and R-based BIO3D package (http://thegrantlab.org/bio3d/). Requirements for data sharing apply to this manuscript. Parameter files are publicly available from http://mackerell.umaryland.edu/charmm_ff.shtml. Molecular structures used are available from https://www.rcsb.org/. Output files are available from the corresponding author upon request.
