# Supplementary material for: A Potential Route of Capsaicin to Its Binding Site in the TRPV1 Ion Channel
Source: J Chem Inf Model. 2022 May 3;62(10):2481–9. doi: 10.1021/acs.jcim.1c01441 (PMC9131452; doi:10.1021/acs.jcim.1c01441)
Supplement: Supplementary file 1 — ci1c01441_si_001.pdf [file ci1c01441_si_001.pdf]

A potential route of capsaicin to its binding site in the TRPV1 ion channel

Carmen Domene,[1,2,3] Leonardo Darré,[1] Victoria Oakes[2], Saul Gonzalez-Resines[2]

[1]Department of Chemistry, King's College London, Britannia House, 7 Trinity Street, London SE1 1DB, UK
[2]Department of Chemistry, University of Bath, 1 South Building, Claverton Down, Bath BA2 7AY, UK
[3]Chemistry Research Laboratory, Mansfield Road, University of Oxford, Oxford OX1 3TA, UK

[#] Corresponding author: C.Domene@bath.ac.uk

(A) **(B)**

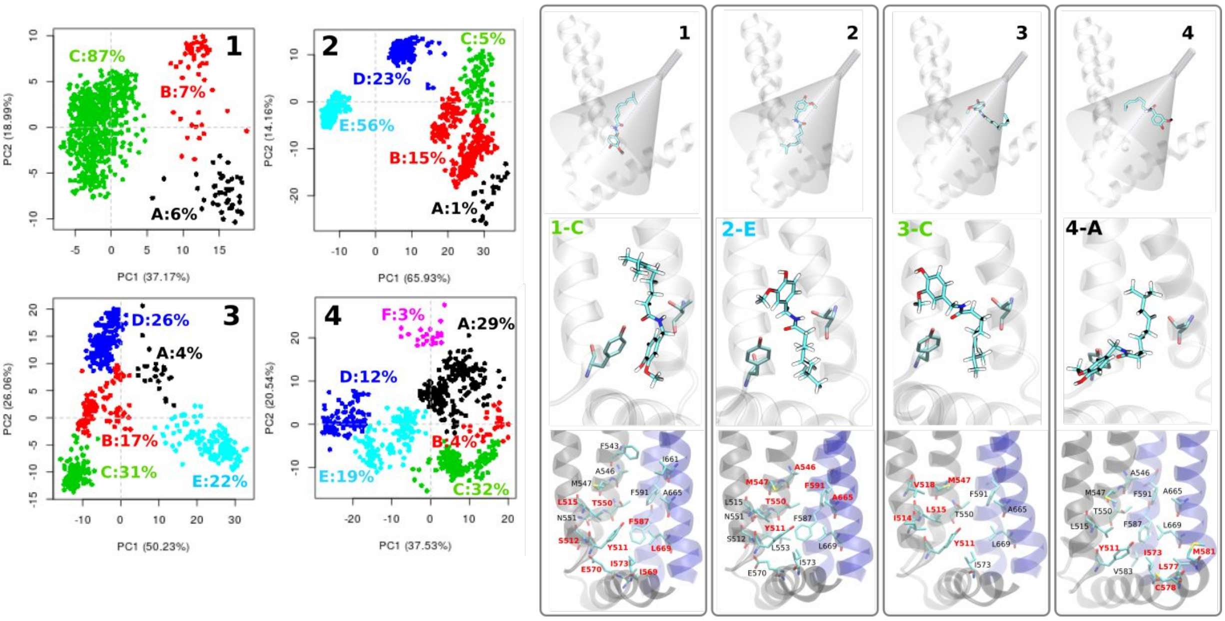

Figure SI 1. Conformational landscape of capsaicin along the pathway from the membrane to the TRPV1 binding site for the simulation were the metadynamics run could not be converged. (A) Scatter plot of the principal components PC1 versus PC2 obtained from the conformations in Cartesian space explored by capsaicin in each high probability region observed in the free energy map. Dots represent (PCA-based) clusters identify for each conformation. Cluster labels and population percentage are indicated. **(B)** Structural diversity along the binding pathway: representative structures from high probability regions in the framework of the binding pocket (for clarity, only helices S3, S4 and S4S5-linker of one out of the four chains are shown) and the funnel-shaped restraint (grey funnel) used to restrict the sampling. Central panel: representative structures of the most populated clusters of high probability depicting part of helices S3, S4 and the S4S5-linker in grey. Residues Y511 and T550 are included for position reference. Residues Y511 and T550 and capsaicin are shown in licorice representation. Bottom panel: main residues in contact with capsaicin for selected clusters. Residues anchoring capsaicin vanillyl ring are in red.
